# Sex and Gender Differences in Emotion Recognition and Theory of Mind After TBI: A Narrative Review and Directions for Future Research

**DOI:** 10.3389/fneur.2020.00059

**Published:** 2020-02-04

**Authors:** Lyn S. Turkstra, Bilge Mutlu, Caitlin W. Ryan, Emily H. Despins Stafslien, Erica K. Richmond, Emily Hosokawa, Melissa C. Duff

**Affiliations:** ^1^School of Rehabilitation Science, McMaster University, Hamilton, ON, Canada; ^2^Department of Computer Science, University of Wisconsin-Madison, Madison, WI, United States; ^3^ThedaCare Regional Medical Center, Neenah, WI, United States; ^4^Central Montana Learning Resource Center Cooperative, Lewistown, MT, United States; ^5^Department of Communication Sciences and Disorders, University of Wisconsin-Madison, Madison, WI, United States; ^6^Department of Surgery, University of Wisconsin-Madison School of Medicine and Public Health, Madison, WI, United States; ^7^Department of Hearing and Speech Sciences, Vanderbilt University, Nashville, TN, United States

**Keywords:** social cognition, gender, sex difference, brain injury, adult

## Abstract

A growing body of literature has examined sex differences in a variety of outcomes from moderate-severe traumatic brain injury (TBI), including outcomes for social functioning. Social functioning is an area in which adults with TBI have significant long-term challenges (1–4), and a better understanding of sex and gender differences in this domain may have a significant clinical impact. This paper presents a brief narrative review of current evidence regarding sex differences in one aspect of social functioning in adults with TBI: social cognition, specifically affect recognition and Theory of Mind (ToM). Data from typical adults and adults with TBI are considered in the broader context of common stereotypes about social skills and behaviors in men vs. women. We then discuss considerations for future research on sex- and gender-based differences in social cognition in TBI, and in adults more generally.

## Introduction

In 2001, a U.S. Institute of Medicine (IOM) report (5) stated that sex-based differences were a priority area for all research on human health. This statement was a change for clinical and basic TBI research, which had focused mostly on men for both epidemiological reasons (higher prevalence in males) and practical reasons (e.g., effects of fluctuating hormone levels). In the years since the IOM report, studies have examined sex differences in a variety of TBI outcomes, including outcomes for social functioning. Social functioning is arguably the area in which adults with TBI have the greatest long-term challenges (1–3, 6, 7). Thus, a better understanding of sex differences in this domain may have a significant clinical impact.

This paper begins with a brief narrative review of research on sex differences in one aspect of social functioning in adults with moderate-severe TBI: social cognition, defined broadly as the processes used to decode the social world (8). The review focuses on two aspects of social cognition that have been studied in TBI: recognition of emotions from facial affect; and Theory of Mind (ToM), the ability to attribute mental states to oneself and others, and use that information to make predictions about others' actions (9). We chose these two aspects of social cognition because they have been linked to broader social outcomes like quality of life and social reintegration, both conceptually (10–12), and empirically (13–20). We consider data from typical adults and adults with TBI, in the broader context of common stereotypes about social skills and behaviors in men vs. women. The remaining sections of the paper discuss considerations for future research on sex-based differences in social cognition in TBI, and in adults more generally.

As defined by the Institute of Medicine, sex is the “classification of living things, generally as male or female based on their reproductive organs and functions assigned by a chromosomal complement;” whereas gender “refers to a person's self-representation as male or female, or how that person is responded to by social institutions based on the individual's gender presentation” [(5), p. 1]. Most of the studies we reviewed focused on biological sex, and we indicate gender where it was clearly defined. We return to the issue of sex vs. gender in our hypotheses about social cognition after TBI. We use the terms “female” and “woman” interchangeably, typically the former as an adjective and the latter as a noun.

## Sex Differences in Social Cognition in Typical Adults

Nowhere are there more profound and enduring stereotypes for men and women than in “social thinking.” The stereotype that women are better at “reading” other people has some empirical support. Several studies have reported a female advantage in emotion recognition in typical adults (21–27), beginning in childhood and persisting throughout life (28–30). Differences generally are small (e.g., accounting for <10% of variance in scores) and are mostly for threat-related affective displays. Analysis of 14,000 samples of written and spoken language showed that women also used more emotion words (e.g., happy, certainty, nervous, and hate) than men, and fewer swear words (31), although again effect sizes were small [i.e., 10–22].

One challenge in generalizing study results to real life is that most stimuli were some version of the iconic six “basic” emotions popularized by Ekman in the 1970s (32). These canonical stimuli do not capture the subtle and dynamic affect displays encountered in everyday social interactions, and women might be better at reading the latter. Consistent with this notion, Hoffman et al. (33) found sex-based differences only for morphed images that were 40–70% of the full facial expression, with no difference for full facial expressions. These findings replicated findings from a previous morph study from the same lab (34) and suggest that more subtle tests might reveal larger sex-based differences.

A second challenge to generalizing results is that participants in prior work were typically given unlimited time to respond. In everyday life, facial and vocal affect displays change in milliseconds (35), a phenomenon Ekman (36) himself exploited in his “lie detection” research, and women may be better at making those quick judgements (24). Again, consistent with that notion, women had higher accuracy scores than men when stimuli were presented at very brief durations (24), identified emotions earlier in the series of morphs than men (34), and overall responded more quickly than men for both morphs and static images (Byom et al., in preparation). Taken together, these results suggest that women are faster at recognizing emotions overall, especially when affective displays are subtle.

By contrast to the literature on emotion recognition, only a few studies have addressed sex differences in ToM or “cognitive empathy” (37). The typical experimental ToM task is a version of the classic False Belief task (38) or Piaget's (39) perspective-taking task, in which the participant must recognize that one actor in a scenario has access to information that the other does not. Most studies have reported no significant difference on ToM tasks between men and women (37, 40–42) or girls and boys [e.g., (43)]; although some have reported trends for better scores in females [e.g., (44)]. ToM findings contrast with those on emotional empathy (feeling the feelings of others), for which women are thought to have an advantage (45).

Taken together, studies of typical adults suggest a female advantage for recognizing emotions in affective displays of others, albeit a small advantage and mostly on subtle or complex tasks. There is no evidence of sex differences in ToM, at least on classic perspective-taking tasks, despite the public perception that women are better at “mindreading.”

## Sex Differences in Social Cognition in Adults With TBI

To identify articles for the narrative review of TBI studies, we searched PubMed, PsychInfo, CINAHL, and Web of Science using the search string: (emotion recognition OR affect recognition) AND (social cognition OR theory of mind) AND traumatic brain injury AND (sex difference or gender difference), with the limits of *human* and *adult*. We excluded review papers and theoretical papers that did not contain data, studies of children, and studies that included men and women with TBI but did not report their scores separately.

The literature search yielded three papers (26, 46, 47) that examined sex differences in social cognition in adults with TBI. Two additional papers reported scores separately for women and men with TBI (41, 42). A third reported sex differences in the context of other findings (48), but the clinical group included participants with etiologies other than TBI, and scores for the TBI subgroup were not reported separately. Thus, that paper was excluded. Of the five studies summarized here, two tested emotion recognition and three tested Theory of Mind. Study characteristics are summarized in Table 1.

**Table 1:** Summary of studies reviewed.

**References**	**TBI group**	**Comparison group**	**Constructs assessed**	**Main findings**
Rigon et al. (26)	53 adults with moderate-severe TBI (28 females)	49 adults (22 females) matched demographically by group	Emotion recognition from static and morphed faces	No significant sex difference on static images; significant group X sex interaction for morphed images, with lowest scores in males with TBI
Turkstra et al. (41)	58 adults with moderate-severe TBI (24 females)	66 adults matched demographically by group	ToM in video vignettes	No significant sex difference
Turkstra (42)	19 adults with moderate-severe TBI (9 females)	19 adults matched for age and sex	ToM in still images and video vignettes	Significant group X sex interaction for still images, with lowest scores in males with TBI; trend toward significant interaction on video vignettes, with lowest scores in males with TBI
Zupan et al. (46)	160 adults with moderate-severe TBI (44 females)	Published norms	Affective empathy and ToM (perspective taking) in written statements	No significant difference in proportion of men vs. women with TBI who scored in the impaired range compared to norms
Zupan et al. (47)	160 adults with severe TBI (116 males)	None	Facial and vocal affect recognition from static images, ToM (emotional inferencing) in movie scenes	Significantly higher scores in women for vocal affect and ToM; trend for women to have higher scores for facial affect

*ToM, Theory of mind; TBI, traumatic brain injury*.

Rigon et al. (26) compared men and women on recognition of both the iconic Ekman-type emotions and also morphing images, which yield accuracy scores according to both emotion type and intensity. The authors found a small but significant female advantage on both tasks. This advantage was independent of emotion type or intensity, injury characteristics such as chronicity and severity, cognitive ability as indexed by neuropsychological test scores, or lesion laterality. Overall, scores of females with TBI were not significantly different from those of age-, race-, and education-matched uninjured peers; whereas males with TBI were significantly less accurate than either uninjured men and women or women with TBI. It is noteworthy that Schmidt et al. (49) found similar results in children with TBI, i.e., higher emotion recognition scores in girls with TBI than boys. The authors hypothesized that this difference might reflect the “small but statistically significant” female advantage in typical development.

Zupan et al. (50) administered two affect tests to compare men and women with TBI: a basic facial and vocal affect recognition test, and a task the authors developed to test inference of emotions from video clips Women were more accurate on two of the three tasks—vocal affect recognition and emotional inference—with small to moderate effect sizes. Contrasting with the findings of Rigon and colleagues, there was no significant sex difference in facial affect recognition, which the authors hypothesized might reflect the prolonged stimulus exposure in their task (i.e., previous studies of typical adults showed that men were as accurate as women when response demands were lower). Interestingly, women were more accurate than men at recognizing fear in faces, sadness in voices, and both fear and sadness from stories; while there were no significant differences in accuracy for angry or happy stimuli. These emotion-specific findings are consistent with overall trends in data on emotion recognition in adults, as fear in particular is difficult to differentiate from surprise or sadness, particularly when participants are asked make judgements early in the temporal evolution of an emotional expression (51).

Léveillé et al. (52) reported emotion recognition scores for male and female athletes with a history of two or more concussions, compared to athletes with no concussion history. This study was initially excluded because the focus of the review was moderate-severe TBI, but the task was similar to the morph task described in Rigon et al. (26), so results might provide an informative comparison. Results were similar to those of Rigon et al.: a main effect of group and a group X sex interaction, with higher scores overall in women and disproportionately lower scores in men with a concussion history. Emotion-specific findings also were replicated, with fear having the lowest accuracy and highest intensity threshold for detection.

Zupan et al. (46) compared men and women with TBI on a self-report measure of perspective taking, in which participants are asked how well they are described by each of a series of statements (e.g., “I try to look at everybody's side of a disagreement before I make a decision”). Scores for both men and women were significantly lower than norms for the measure, a comparable percent of participants of each sex were classified as “impaired” according to those norms, and there was no significant difference in total scores between men and women. Close others also rated each participants' empathy. Men significantly under-rated their problems relative to ratings of their close others, whereas there was no significant difference between self- and others' ratings for women. Seventy-eight percent of all close others for both groups were women, a potential source of observer bias we will return to later in this paper.

Turkstra (42) compared men and women with TBI to uninjured peers on a ToM test, and replicated the study in a subsequent sample (41). Participants watched brief video vignettes of social interactions and made ToM judgements about actors in the videos. In the initial study, there was a significant group X sex interaction, with women performing better overall and disproportionately lower scores in men with TBI. The follow-up study, however, showed no significant effect of sex or group X sex interaction, although women had higher scores than men. It is not clear why results of the two studies differed. In both studies, TBI and uninjured comparison participants were matched for age, race, education, and sociodemographic factors; participants were drawn from the same general pool of Midwestern adults; and adults in both studies had moderate-severe injuries. It is not possible to directly compare cognitive status between the two samples as the tests differed, but in both cases participants with TBI had significantly lower scores than uninjured peers. Future research may clarify whether there truly is no difference or if a difference is only present on certain tasks.

Overall, while the rationale for studying sex-based differences in social cognition after TBI is strong, the literature is sparse, results are mixed, and effect sizes are generally small. In the remainder of this paper, we discuss directions for the future and some reasons why research in this area is challenging.

## Considerations for Research on Sex-Based Differences in Social Cognition in TBI

The current state of the science on sex-based differences in social cognition, along with our own reflections on sex and gender in social cognition, have led us to a few considerations for future work in this area.

### Social Cognition May Be Related to Gender as Well as (or Instead of) Biological Sex

Up to this point we have focused on sex (the biological construct), but gender also may play a role in social cognition. Gender, while typically rooted in biology, is a social construct. It includes “how you, in your head, define your gender, based on how much you align (or don't align) with what you understand to be the options for gender” (53). Thus, while sex is typically binary, gender is on a continuum and may be fluid. Gender includes one's internal representation in relation to gender norms, how one expresses gender through outward appearance, and the roles one takes in social contexts, all of which may be fluid and dynamic (53).

We began thinking about gender as a factor in outcome in part because of the variability within each sex in our studies [e.g., (42)] and others' [e.g., (54, 55)]; and in part because of evidence of gender differences in cognitive functions linked to social cognition, particularly executive functions (EFs) (56, 57). Research on sex differences in EFs has been mixed but results from self-report EF scales have hinted at gender effects. For example, Norvilitis and Reid (58) asked 234 university students to complete a self-assessment of gender, the Bem Sex-Role Inventory (BSRI) (59), discussed in detail below) and the Executive Function Scale (60), an EF self-report measure. The authors found that, controlling for biological sex, masculine BSRI scores were positively correlated with self-reported EFs. Similarly, Turkstra et al. (61) administered the BSRI and the Behavior Rating Scale of Executive Function-Adult version (BRIEF-A) (62) to 53 adults with TBI (23 females) and 49 uninjured adults (29 females), and found that a significant amount of variance in BRIEF scores in both groups were accounted for by self-reported masculinity (t = −4.57, *p* < .001), but not biological sex (t = 0.96, *p* =.33) or self-reported femininity (t = −0.41, *p* = 0.68). These early findings raised questions about the role of gender as a predictor of behavioral outcome after TBI, and also how self-identity as a man or woman might differ from self-described gender role in social contexts.

Our first barrier to studying gender was that the terms are often used interchangeably. Indeed, confusion of the terms sex and gender is a key barrier in research on sex-based differences in general (5, 63). In TBI, it is almost impossible to identify the influence of gender on outcomes because the terms are used so inconsistently in the scientific literature. To illustrate, we searched PubMed publications for the past 5 years using the keywords “gender,” “social communication,” and “human.” We retrieved 85 articles, and in all but three the word *gender* referred to biological sex. Some authors stated that they categorized sex using self-report questionnaires or hospital records, but most did not state their methods, which likely means that they either based their categorization on participants' responses to a multiple-choice question (e.g., *Circle one: M F*) or judged sex based on appearance, which is linked to gender not sex (53). It may be, then, that one factor confounding results in the literature on “sex-based differences after TBI” is the conflation of biological sex and gender.

Although there have been no studies of gender and social cognition, a few researchers have examined gender identity related to social functioning after TBI (64–68), and findings are informative for future social cognition studies. In a study of 33 males in the chronic stage after TBI (65), Schopp and colleagues found a significant correlation between some aspects of self-reported conformity to masculine gender roles (e.g., valuing winning) and outcomes such as earning, but most gender role variables were not significantly related to outcome variables. The highest correlation was between earnings post-injury and self-reported conformity to male violence norms, defined as the “tendency to utilize or value violence and beliefs that violence is sometimes required and justified” [(65), p. 1158]. The findings must be interpreted with caution, however, as results for 13 gender variables were correlated with four outcome variables in a sample of 33 participants, thus the analysis had a high risk of Type I error (finding differences where none exist).

Gutman and Napier-Klemic (69) conducted in-depth interviews with two men and two women with TBI to explore gender identity and gender role changes post-injury. The women reported less impaired internal gender identification while the men reported a sense of inadequacy in their gender role. In both cases, perceptions about gender role appeared to be related to the ability to participate in pre-injury activities that defined their masculine or feminine role. The authors interpreted this relationship as lack of participation causing perceived role changes; but impairments in social cognition skills needed for these “gendered” activities also could have been a contributing factor.

Alston, Jones and Curtin (70) conducted in-depth interviews of 11 women and 21 men with TBI in Australia. Narratives emerging from the in-depth interviews of women included themes related to power, control, the body and self-image, and the gendered nature of caretaking. The authors noted that outcomes after TBI reflected broader gender-linked trends in society, e.g., women reported increased self-consciousness about their bodies and body image post-injury, almost half of the women reported being a victim of financial abuse by people close to them, and only four of the women reported being cared for by a family member. These results show the impact of societal expectations and the reality of gender influences on everyday life.

While gender identification is a critical variable in research, measuring it has proven to be a challenge. The BSRI (59) is perhaps the most consistently used tool, and its challenges illustrate the broader challenges of measuring gender in research. The BSRI is based on social constructs of femaleness or maleness, mostly in Western culture, and reflects links between the experience of gender and the person's social context (71). The premise underlying the BSRI is that each of us have both masculine and feminine personality characteristics and that gender-typing depends on the balance between these characteristics. The BSRI is comprised of a list of 60 personality characteristics grouped into three categories based on ratings by 100 undergraduate students in the 1960s (50 self-identified as women, 50 as men): 20 masculine characteristics, 20 feminine characteristics, and 20 neutral characteristics. The author categorized a characteristic as masculine or feminine if male and female judges agreed that it was more desirable for one sex or the other. Masculine characteristics include items such as *assertive* and *strong personality*, and feminine characteristics include items such as *compassionate* and *soft spoken*. Neutral items were those independently judged by men and women to be no more desirable for one sex than the other, and judged as equally desirable by men and women raters. Of the neutral group, 10 items were rated as highly desirable for anyone (e.g., *tactful, friendly*) and 10 were rated as highly undesirable (e.g., *conceited, unpredictable*).

To administer the BSRI, the experimenter asks respondents to indicate how well each characteristic describes them on a seven-point scale from 1 (never or almost never true) to 7 (always or almost always true). Each person receives a masculinity score, a femininity score, and an androgyny score. The androgyny score is calculated as the Student's *t*-ratio for the difference between the masculinity and femininity scores, i.e., the absolute difference between masculinity and femininity normalized with respect to the standard deviations of that participant's masculinity and femininity scores. Using the androgyny score, the individual is typed as masculine, feminine, or androgynous.

The first potential critique of the BSRI is that it is extremely dated. As one might expect, a 2006 study of undergraduate students did find some changes (72): stereotypic desirable behaviors for men again aligned with traditionally masculine traits such as *has a strong will, very active*, and *knows the way of the world*; but socially desirable behaviors for women included not only traditionally feminine traits such as *very understanding of others, very considerate of others*, and *very aware of feelings of others*, but also traditionally masculine traits such as *strong, independent*, and *enjoys a challenge*. Similar findings were reported by in three other studies at around the same time (73–75). These results suggest that male stereotypes have changed relatively little over time, at least until the early 2000s, whereas female stereotypes have expanded to include both masculine and feminine traits.

The BSRI, like other gender role and gender identity scales that rely on *a priori* social judgments [e.g., the Personal Attribute Questionnaire (76, 77)], also has been criticized for limitations in construct validity (78), and there has been debate about the scale factor structure, particularly the masculine factor (79–81). Nevertheless, the BSRI remains the most common measure of gender role in health-related research and is one metric to consider when evaluating gender. Whatever the tool used, measuring gender is clearly different from measuring biological sex.

### To Capture Sex-Based Differences in Social Cognition, We Need More Sensitive and Realistic Experimental Tasks and Larger, Representative Samples

Results of emotion recognition research strongly suggest that most research stimuli do not include the subtle and rapidly changing affect cues for which women might have a marked advantage. Research is critically needed in this area, not only to characterize sex differences but also predict how social cognition impairments will manifest in everyday life for people with TBI. Video assessments such as The Awareness of Social Inference Test (82) and morph tasks such as the Emotion Recognition Test (22, 83) are a step toward analysis of in-the-moment affect recognition, but finer-grained measures and more complex stimuli are needed.

Sex-based differences also may be missed when samples include too few women or are generally underpowered. Women often are unrepresented in studies of TBI and some samples are composed entirely of men [e.g., Vietnam Head Injury Project (84)]. When samples are not well-balanced for sex, it may be difficult or impossible to detect meaningful and statistically significant sex differences. Even when samples do contain men and women, the data are seldom stratified by sex. Despite the well-documented heterogeneity in deficit profile and outcome following TBI, samples in TBI studies remain small. To detect reliable sex differences in social cognition, or in any domain, samples need to be considerably larger and efforts must be made to have more balanced samples with regard to sex.

### Most Clinical Data Are From Self-Ratings, Which Are Prone to Stereotype Bias

Social communication, by definition, is communication in a social context and thus is subject to stereotypes about roles and behaviors. One's concept of maleness and femaleness is used to create standards of masculine and feminine gender-roles against which one perceives, categorizes, and evaluates their own behavior and personality and the behavior and personality of others (59), and this relationship is as true for social behaviors as it is for other domains. In other words, it's not just the person's ability but also what society expects of that person based on gender norms. A well-known example from the popular press is the observation by Tannen (85) that women use social interactions to build consensus or share thoughts and feelings, show more listening behavior and less interrupting in conversations, and show more self-disclosure and openness in their talk. While Tannen's observations were not experimentally derived, the general patterns have been confirmed empirically [e.g., (86)], and the notion of sex-based differences in communication “style” has become part of our culture. Thus, a person with TBI has a double challenge: to perform according to his or her own gendered expectations and to meet expectations of others.

To explore how expectations *of* and *for* men and women might differ today, Stafslien and Turkstra (under review) asked 68 university undergraduates (34 self-identified as women, 34 as men) to identify acceptable behaviors for men and women using the LaTrobe Communication Questionnaire (LCQ), a well-validated questionnaire for evaluating social communication in adults with TBI (87). Participants were asked to rate how much of a problem each LCQ behavior would be if a woman displayed it vs. if a man did. Items were rated as not a problem at all (1), sometimes a problem (2), often a problem (3), or always a problem (4). Mean scores for each item were calculated for women vs. men raters. Items with mean ratings of 2.0 or higher were considered to indicate a problem behavior, and we compared items with high mean scores between male and female raters for the “if a woman did it” and “if a man did it” versions of the LCQ.

Two findings of the Stafslien and Turkstra study were notable. First, male and female raters agreed on six LCQ items that were problems for anyone, male or female (e.g., giving inaccurate information, not knowing when to talk and when to listen, not putting ideas together in a logical way). These items correspond to typical social communication problems in adults with TBI, supporting the ecological validity of the LCQ. Two items were rated by both men and women as problematic if shown by a woman but not a man (using vague or empty words or repeating oneself in conversation), and one if shown by a man but not a woman (saying something rude or embarrassing). Second, women raters identified 23 items overall as problematic if shown by either a man or a woman, and men identified 17. That is, women appeared to have less tolerance for violations of common social behaviors. These results suggested that standards for social cognition might be higher for women, particularly for women who display socially stereotypical feminine behavior, and that women might be harsher judges of social behavior in others. They also raise questions about bias in research and clinical assessment results when “other” raters are caregivers, who most often are women [e.g., (88)].

## A Hypothesis for Future Study

Current data suggest that women have a small but significant advantage on social cognition tasks, an advantage that is most evident when stimuli are subtle and complex. There also are hints that men may be more vulnerable to TBI-related impairments in social cognition, but results are inconsistent. Societal expectations play a critical role in this relationship, particularly given evidence that social stereotypes about sex differences far exceed effect sizes in empirical studies.

**Figure 1:**
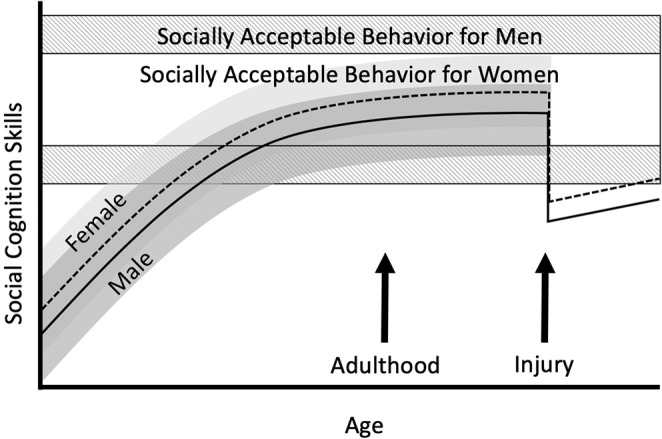
Hypothesized relationships among social development, societal expectations, and effects of TBI in adulthood for men and women.

We propose that social cognitive abilities and societal expectations interact over time in development and after injury, as shown in Figure 1. Women are shown as having a slight advantage in social cognition from childhood, and perhaps more “resistance” to TBI effects (at least for the types of stimuli typically used in research), but the range of acceptable behavior for women is narrower than the range for men; thus, women might see themselves—and others around them might see them—as less skilled in traditional female roles. Men are shown as starting out with slightly less skill in social cognition, and again potentially being more vulnerable to TBI effects, but this is offset somewhat by the broader range of acceptable behavior for men. The result of this interaction might be a difference between one person's test scores, self-ratings of social functioning, and ratings by close others. We have omitted gender from the figure because there are no data, but emphasize that gender must be considered in this model.

The model in Figure 1 depicts injury sustained in young or middle adulthood. Equally critical are effects of injury in childhood and in older adulthood. The importance of understanding sex differences at these life stages is supported by emerging evidence of pediatric TBI effects on social cognition and communication (89, 90), and sex differences in outcome from pediatric TBI more broadly; and evidence of persistent social problems in older adults (91).

## Implications for Clinical Intervention

Current gaps in knowledge have several implications for clinical intervention. First, raters must consider their own potential bias in assessment, such as judging behaviors based on social stereotypes (e.g., “that's typical male behavior”). Second, clinicians must consider not only test or questionnaire results, but also the patient's gender identity—i.e., alignment with gendered roles of that person's social context. Patients might not spontaneously offer those perceptions, but they are important for treatment. Third, as part of patient-centered care, it is important to know what that individual's gender role, identity, and expression were pre-injury, as that will influence treatment goals and expectations for that person. Finally, it is possible that women and men (or relatively male or female persons) respond differently to treatment, which may have confounded previously reported treatment study results. This was the case for Babbage et al. (92), who initially found no significant benefit of a story-based affective intervention in adults with TBI (93), but later discovered that the subgroup of women did indeed show treatment effects. The potential interaction of TBI and sex (or gender) in treatment must be addressed in future studies.

## Conclusions

In summary, social functioning may be the most common and consequential area of long-term deficit for individuals with TBI, affecting all aspects of life. It is also a common target for treatment. Understanding how sex and gender play a role in social functioning, including social cognition, will advance our knowledge about social functioning after TBI, and help identify meaningful and effective intervention methods.

## Author Contributions

LT is the senior researcher on this team, led much of the work cited, and led writing of the manuscript. BM was co-investigator on the work cited and analyzed sex-based data for studies cited. CR completed the review of sex differences in emotion recognition. ED conducted the research on perceptions of sex differences and completed the review of sex differences in perception of social behavior. EH and ER completed the review of sex differences in executive functions, and introduced the concept of gender identity vs. expression. MD was co-investigator on the work cited here and led analysis of sex differences in emotion recognition after TBI.

### Conflict of Interest

The authors declare that the research was conducted in the absence of any commercial or financial relationships that could be construed as a potential conflict of interest.
